# Supplementary material for: Are carbon emissions trading and green financial instruments synergistic? -Comprehensive quantitative research based on content analysis
Source: PLoS One. 2024 Mar 7;19(3):e0298601. doi: 10.1371/journal.pone.0298601 (PMC10919601; doi:10.1371/journal.pone.0298601)
Supplement: S1 Data — (ZIP) [file pone.0298601.s001.zip › Data/地级市Z_F工作报告.zip!/║╙▒▒/╟╪╗╩╡║╩╨/└┤╫╘╣1⁄2╓┌║┼í╛┬φ┐╦╩2╛▌═°í┐_2018.docx]

各位代表：

现在，我代表市人民政府，向大会报告政府工作，请予审议，并请市政协委员和列席会议的同志提出意见。

一、2017年工作回顾

过去的一年，是秦皇岛发展史上不寻常、不平凡的一年，是发展理念实现重大转变、各项事业取得重要成就的一年。在市委的坚强领导下，全市上下以习近平新时代中国特色社会主义思想为指导，以迎接庆祝学习贯彻党的十九大为动力，迎难而上、拼搏进取，稳增长、促改革、调结构、惠民生、防风险，全年实现地区生产总值1506.01亿元，比上年增长7.3%，增速居全省首位，全部财政收入230.49亿元，增长13.3%，城乡居民人均可支配收入分别达到32795元和12563元，均增长8.1%；成功摘取全国文明城市桂冠，精彩承办第二届省旅发大会，圆满实现“历史最好”暑期目标，城市环境品质、干部队伍作风、市民精神风貌焕然一新，沿海强市、美丽港城和国际化城市建设迈出坚实步伐。

一年来，我们重点抓了以下七方面的工作：

（一）深入推进供给侧结构性改革，经济发展质量效益同步提升。坚持去旧、添新、增特、提质并举，压减炼铁产能525万吨、炼钢产能353万吨、水泥产能201.5万吨、平板玻璃产能240万重量箱，上半年即超额完成省定年度压减任务，预计全年万元生产总值能耗降低5%左右。促进发展动能换挡提速，发挥市投资促进和企业服务中心作用，点对点、面对面解决项目建设、企业发展中的问题，中秦兴龙产业园建成投产，威卡威汽车零部件北方基地试生产，京能热电等重点项目开工。完成城乡建设项目投资706.44亿元，增长13.4%；完成工业投资230.19亿元，增长10.7%，其中工业技改投资增长20.8%；高新技术产业投资增长21.9%。产业结构稳步优化，动能转换逐步加快，创建全国质量强市示范市通过验收，在京津冀地区第一个获此殊荣。规模以上工业完成增加值376.93亿元，增长5.8%，装备制造业、高新技术产业增加值占规模以上工业的比重分别达到41%和35.7%，规模以上工业企业实现利润增长1.27倍，亏损企业亏损额下降54.2%，工商税收增长18.4%。服务业增加值增长9.7%，对经济增长贡献率达到68.1%。以筹办第二届省旅发大会为契机，用30亿元政府基础设施投入撬动了300亿元社会资本，打开山海旅游景观走廊，贯通沿长城旅游环线，秦皇山海康养旅游度假区初具规模。实现旅游总收入658.29亿元，增长32.9%。山海关景区恢复5A级景区质量等级。港口货物吞吐量2.45亿吨，集装箱55.9万标箱。社会消费品零售总额775.18亿元，居民消费价格指数101.7%。新增省级现代农业园区、休闲农业示范区8家，我市成为全省唯一农业可持续发展试验示范市。

（二）全面实施改革创新，发展活力和内生动力持续释放。坚持重点领域改革全面发力、多点突破、纵深推进，在全省率先推行城市管理综合执法、海洋和渔业管理体制改革，积极推进行政执法三项制度试点改革。市、县区两级行政审批局挂牌运行。市级经营性国有资产集中统一监管改革稳步实施，耀华集团企业重组取得重大进展，职工分流安置圆满完成。农村土地确权登记基本完成。供销社综合改革全面推进。国有林场改革按期完成。坚持将科技创新作为增强动力的关键点，实施科技型中小企业成长计划，科技型中小企业达1968家；全省首家“外国院士工作站”在我市挂牌成立，院士工作站达16家，特邀院士工作站达10家，省级以上工程技术研究中心、产业技术研究院、重点实验室分别达24家、6家和20家，市级以上众创空间达25家，万人发明专利拥有量7.1件。成功入选全国新一轮“十三五”海洋经济创新发展示范城市。坚持将金融风险防控作为重要保障，实施互联网金融风险和化解不良贷款专项整治，有效处置了一批非法集资案件，金融机构贷款不良率比年初下降0.69个百分点，秦皇岛银行完成增资扩股，昌黎、抚宁、卢龙、青龙成立村镇银行，在多层次资本市场上市企业达72家。

（三）积极融入协同发展战略，开放格局进一步打开。全力推进北戴河生命健康产业创新示范区建设，生态、交通等14个专项规划编制完成，成功申列国家健康旅游示范基地；国际康养旅游中心完成六大中心建设，德国潘纳茜诊疗中心成为全省第一家中外合资医疗机构，索米克等10个尖端机构入驻。务实承接非首都核心功能疏解和京津产业转移，与京津对接合作项目达128项，与北京西城区建立战略合作关系，与中建材、华大基因等企业集团合作进展顺利，北戴河肿瘤医院、秦皇岛广济医院开工建设，清华大学秦皇岛智能装备研究院、北京化工大学秦皇岛环渤海生物产业研究院等11个项目竣工；与北京实验二小、北京金融街集团、赛伯乐集团等合作取得实质进展。以创新机制开展招商引资集中攻坚行动，签约引进了一批产业链配套项目，实际利用外资10.22亿美元，增长13%。开展重点外贸企业“一对一”帮扶行动，实行出口1美元补贴1分钱政策，进出口总额338.19亿元，增长16.7%，其中出口215.69亿元，增长10.7%。秦皇岛港成为国家进境粮食和水果指定口岸，开通至菲律宾达沃直航航线，流失多年的香蕉贸易又在我市重现。

（四）坚持绿水青山就是金山银山，生态文明建设亮点纷呈。把生态作为立市之本和核心竞争力，制定实施大气污染综合治理“1+1+18”政策体系，淘汰燃煤锅炉1066台，提标改造锅炉56台，完成8093户气（电）代煤改造，超额完成省定任务。整治“散乱污”企业944家，推广新能源汽车2981标准车，PM2.5平均浓度下降4.3%。深入推进河长制，加强饮用水源地保护，集中整改污水处理厂19家，关闭搬迁畜禽养殖场933家。市域河流水质功能区达标率明显提升，饮马河退出劣V类水质。近岸海域水质功能区达标率100%，海水浴场水质一类标准比例达99.4%。青龙湖等湿地修复加快推进。我市被国家海洋局确定为湾长制试点城市。修复责任主体灭失矿山33个，整治持证露天矿山94个。严肃查处了一批环境违法案件。园博园建设和栖云山生态修复扎实推进，完成14座公园游园建设改造和22条道路绿化工程。植树造林51.6万亩，森林覆盖率预计达到51%。

（五）全力开展全国文明城市创建，城市形象、品质和功能显著提升。坚持全员、全域、全时抓创建，精耕细作城市治理。以国际标准开展新一轮城市总体规划编制，编制海绵城市、停车设施等7个专项规划，“多规合一”信息平台建成运行。我市被国土资源部列入新一轮土地利用总体规划编制试点城市。着力提升城市综合承载能力，燕大校区、军安小区等区域纳入城市区集中供热管网，北戴河所有小区实现新能源集中取暖，城市集中供热普及率达95%；北戴河西部水厂建成投用；新建污水处理厂3家，第四污水处理厂等4家污水处理厂完成升级改造，全市城镇污水处理全部达到一级A标准；北戴河机场二期通过验收，开通国际国内航线17条；北戴河机场快速路主体完工，宁海大道、祖山大道全面建成，京秦高速二通道开工建设，卢新支线、102国道市区绕城通道前期工作顺利推进。百年地方铁路与国铁正式接轨，承秦铁路列入国家中长期铁路网规划。狠抓城市环境治理，累计整治“双违”1587.62万平方米，台账内“双违”全部清零。在全省率先实现数字化城管向智慧城管升级。以务实行动加快新型城镇化进程，开展县城建设三年攻坚行动，实施重点项目76个，完成投资26亿元。所有县城实现环卫清扫全覆盖，垃圾无害化处理率达到100%。卢龙县国家新型城镇化综合试点和石门镇中国特色小城镇建设积极推进，北戴河新区医疗康养旅游小镇等6个特色小镇入围省首批特色小镇。预计常住人口城镇化率达57.9%。

（六）着力增进民生福祉，人民生活不断改善。坚持以人民为中心的发展思想，民生支出占一般公共预算支出的比重达到85.6%，年初承诺的20件民生实事全部兑现。将扶贫开发作为一号民生工程，深入开展驻村帮扶行动，减贫3.4万人，青龙满族自治县综合贫困发生率降至1.13%。持续提高公共服务质量水平，改造提升老旧小区215个、背街小巷216条，改造棚户区住房18533套。农村饮水安全巩固提升工程惠及3万人口。城镇新增就业5.89万人，登记失业率2.88%。城乡最低生活保障、居民基础养老金标准和企业职工养老金水平进一步提高。在全省率先实现城乡居民基本医疗保险市级统筹，大病保险实现全覆盖。19家城市公立医院全部取消药品加成，9个医联体组建完成，家庭医生签约人数达110.9万。市博物馆开工建设，国家语言文字推广基地建成投用，公共文化服务体系示范区建设保持全省前列。成功举办国际马拉松等重大赛事。开展“啄木鸟”行动等安全生产大检查活动，强化食品药品安全整治，加大社会治安综合治理力度，加强和创新社会治理，社会保持和谐稳定。

与此同时，我们还聚焦人民日益增长的美好生活需要，认真倾听民心民意，以改革的思维、担当的精神、创新的办法，解决了一批百姓关心关注的热点难点问题。围绕破解房地产开发遗留问题，按照“两分离”原则，化解遗留问题159个，149个“办证难”问题基本解决，超过10万户居民住房具备不动产登记条件。困扰多年的海港区开滦路、交建里、西部四村开发建设难题取得重要进展。围绕破解教育发展不均衡问题，大力实施“全面改薄”工程，累计竣工学校331所，4个教育集团正式成立，主城区12所中小学建设取得突破性进展。新改扩建幼儿园25所，撤并教学点52个，青龙营养餐工程惠及4.29万学生。围绕破解城市交通拥堵问题，打通山东堡路等断头路9条，新开通公交线路11条，优化调整微循环线路11条，完成抚宁区公交化改造；对城市区69个路口进行改造，路网通行能力整体提升33.5%，实现全省最安全、最畅通、最有序、最文明城市交通环境目标。围绕破解乡村环境差问题，借助承办省旅发大会东风，推动7个县区23个乡镇394个村环境面貌发生根本变化，2041个村建立农村垃圾治理市场化运作机制，打造美丽乡村建设省级重点村202个，农村生产生活环境显著改善。

（七）推进政府管理创新，行政效率、服务水平明显提高。深化“放管服”改革，12批558项上级取消下放行政权力事项全部衔接落实，63项市级行政权力点对点下放给秦皇岛经济技术开发区、北戴河新区和海港区开发区。在全省率先实施“三十三证合一”登记制度，全面实施企业和群众到政府办事“最多跑一次”改革，承诺事项达到397项。加强重点领域立法，全面推进行政执法公示、执法全过程记录和重大执法决定法制审核。自觉接受人大法律监督、工作监督和政协民主监督，办理全国、省和市人大代表建议、政协提案590件，按时办复率和规范化率均达100%。全面推进政府系统从严治党，认真履行主体责任，严格落实中央八项规定精神，驰而不息纠正“四风”，政府系统工作人员思想观念、工作作风发生新变化。

同时，我们扎实开展国防动员、双拥共建和优抚安置工作，积极推进军民融合发展，驻秦部队和广大民兵在森林防火等急难险重任务中作出了突出贡献。民族宗教、新闻广电出版、外事侨务、人民防空、方志档案、气象地震、审计、统计、援疆、妇女儿童、老龄、残疾人等事业都有了新的发展和进步。

各位代表，奋进的历程不同寻常，取得的成绩殊为不易。这是市委总揽全局、正确领导的结果，是市人大、市政协及社会各界大力支持的结果，是市政府历任组成人员团结奋斗，各县区、各部门和全市人民共同努力的结果。在此，我代表市政府向全市人民，向人大代表、政协委员，向各民主党派、工商联、无党派人士和人民团体，向驻秦人民解放军、武警官兵和公安政法干警，向中省直机关驻秦单位，向关心支持秦皇岛发展的海内外朋友们，致以崇高的敬意和衷心的感谢！

各位代表，安不忘危，喜不忘忧。我们清醒地认识到，我市发展中还存在许多深层次矛盾和问题，还远远未到歇脚松气的时候，没有任何骄傲自满的理由。一是综合实力不强。地区生产总值、一般公共预算收入、固定资产投资、规模以上工业增加值等部分指标，尽管省内排名靠前但没有实现预期目标，大项目特别是产业类项目少，经济总量与同期同类沿海开放城市相比还有很大差距。二是发展质量不高。产业大多处于价值链低端，创新能力不强，高品质、高附加值产品缺乏，知名品牌少，技术创新成果不多，新兴产业发展规模尚不能有效填补去产能空缺。三是生态环境改善任务艰巨。空气质量优势相比周边地区日渐缩小，水污染源未有效根除，海洋环境保护力度需进一步加大，部分区域、部分企业、部分行业生态环境问题依然突出。四是发展环境亟待优化。文明城市尚未形成长效化、常态化机制，许多领域改革尚需深化，开放水平和层次尚待提高，服务市场主体意识不到位，投资创业还有不少显性、隐性门槛。五是风险隐患不容忽视。市、县区政府债务偏高，违法违规金融活动时有发生，安全发展面临不少挑战。六是全面建成高质量小康社会还有很多短板要补。脱贫攻坚工作相比“稳实准长”要求还有一定差距，教育、医疗、就业、养老、文化等方面还不能很好满足人民群众的需要，社会建设还面临不少难题。七是政府自身建设尚需加强。一些政府部门不担当不作为、基础工作不扎实、政策措施不精准，少数政府工作人员观念陈旧、作风不实、能力不足，“四风”问题和消极腐败现象不同程度存在。这些问题如果得不到有效解决，我们的发展制约就难以突破，发展空间就难以打开，发展动力就难以为继，发展活力就难以增强，发展成果就难以共享，全面建成高质量小康社会以及沿海强市、美丽港城和国际化城市的目标就难以实现。我们必须常怀忧患意识，突出问题导向，勇于攻坚克难，加快推动经济社会实现更高质量、更有效率、更加公平、更可持续发展。

各位代表，积力所举，无往不胜；众智所为，无所不成。过去一年的实践充分证明，有创城精神、旅发速度、暑期标准为引领，有敢干事、想干事、能干事的干部队伍作保障，只要我们始终保持永不懈怠的精神状态和一往无前的奋斗姿态，就一定能够在全面决胜小康社会新征程中战胜各种风险和挑战，取得新的更大的业绩！

二、2018年政府工作总体要求

党的十九大作出了中国特色社会主义进入新时代的重大判断，省委九届六次全会吹响了河北新时代发展的号角。站在新的历史起点上，市委十二届二次全会进一步描绘了秦皇岛未来发展的宏伟蓝图：到2020年，秦皇岛要全面建成高质量小康社会，生态环境、城乡统筹、开放水平、城市文明走在京津冀城市群前列；到2030年，建成沿海强市、美丽港城，提前基本实现社会主义现代化，发展质量、改革动力、人民生活、社会治理能力、管党治党水平处于全国前列；到2040年，初步建成一流国际旅游城市，力争成为社会主义现代化强市，实现秦皇岛发展质量和城市品质的跨越提升。这三个阶段在方向上与党的十九大精神一致，在目标上与省委省政府提出的全面建成高质量小康社会和一流国际旅游城市要求吻合，体现了秦皇岛作为沿海城市走在全国前列的志向和担当。

各位代表，今年是贯彻党的十九大精神的开局之年，是改革开放40周年，是决胜全面建成小康社会、实施“十三五”规划承上启下的关键一年，做好今年经济社会发展各项工作意义重大。我们必须进一步“找准方位、明确目标，解放思想、更新观念，抢抓机遇、苦干实干”，在新的发展征途上赢得主动、赢得优势、赢得未来。

今年政府工作的总体要求是：高举中国特色社会主义伟大旗帜，全面贯彻党的十九大、省委九届五次六次全会、省“两会”和市委十二届二次全会精神，以习近平新时代中国特色社会主义思想为指导，坚持稳中求进工作总基调，坚持新发展理念，紧扣我国社会主要矛盾变化，按照高质量发展的要求，统筹推进“五位一体”总体布局、协调推进“四个全面”战略布局，深入实施生态立市、产业强市、开放兴市、文明铸市发展战略，深化供给侧结构性改革，加快建设现代化经济体系，坚持创新竞进、协同融合、改革开放、转型升级、提质增效、改善民生、优化环境，推动质量变革、效率变革、动力变革，促进经济社会持续健康发展，奋力开启新时代建设沿海强市、美丽港城和国际化城市新征程。

实现上述目标，必须始终牢牢把握高质量发展这一根本要求，将创新作为高质量发展的第一动力，深入实施创新驱动发展战略，加快推进以科技创新为核心的全面创新，不断提升科技进步贡献率，抓好企业家、科技人才、党政干部“三支队伍”建设，提高全要素生产率；将协调作为高质量发展的内生特点，深度融入京津冀协同发展，加快发展“四大两特”产业，大力实施乡村振兴战略，着力解决发展中的不平衡不充分问题，做大做强做优港口经济、城市经济、县域经济；将绿色作为高质量发展的普遍形态，持续加力治污染，系统推进生态保护和修复，加快形成绿色生产和生活方式，推进生态与产业深度融合，促进人与自然和谐发展；将开放作为高质量发展的必由之路，以全球视野思考谋划新时代秦皇岛对外开放新思路，主动融入“一带一路”，在开放的范围和层次上进一步拓展，在改革开放的质量水平上实现新跃升；将共享作为高质量发展的根本目的，牢固树立以人民为中心的发展思想，时刻将百姓的安危冷暖放在心上，不断满足群众个性化、多样化、日益增长的美好生活新需求，努力使广大人民群众充分享受高质量小康社会成果。

知之非艰，行之惟艰。推动实现高质量发展，不仅需要理念上转变，更需要行动上自觉、制度上配套、能力上跟进。必须做到知行合一。以高质量发展为导向，建立健全科学的指标体系、政策体系、标准体系、统计体系、绩效评价、政绩考核，创建和完善制度环境；以制度建设为依托，引导各级对照指标标准找差距、补短板、增动力，使高质量成为全市上下的自觉行动和目标追求。必须坚持质速并重。坚持质量第一、效益优先，遵循经济发展规律，把高质量发展贯穿于社会发展各领域、全过程；准确把握秦皇岛发展阶段性特征，保持战略定力，坚定前进信心，促进存量优化、增量扩张，为高质量发展厚植根基、注入动力。必须注重点面结合。坚持问题导向，以顶层设计的精准提高政策指向的精准，以措施的针对性提高工作的时效性；坚持因势利导，综合考虑改革力度、发展速度和社会承受度，加快实现向高质量发展的根本转变。

三、2018年目标任务和主要工作

今年经济社会发展主要目标是：地区生产总值增长7.5%左右；一般公共预算收入增长8%；规模以上工业增加值增长6%；固定资产投资增长8%；服务业增加值增长10%；社会消费品零售总额增长10%；实际利用外资增长8%；进出口总值增长10%，其中出口增长8%；PM2.5年均浓度下降6.8%，化学需氧量、二氧化硫、氨氮、氮氧化物减排完成省定目标任务；城镇居民和农村居民人均可支配收入分别增长7.8%和8.1%；万元生产总值能耗下降4%左右；居民消费价格指数涨幅控制在3%以内；城镇登记失业率控制在4%以内。这些目标的设定，体现了高质量的发展导向，结合了我市发展阶段性特征，有利于经济社会发展提速增效、行稳致远。

围绕上述目标，重点做好以下八方面工作：

（一）坚决打好三大攻坚战。中央确定的三大攻坚战，是推动高质量发展、实现稳中求进、决胜全面建成小康社会的重要举措，必须全力以赴打好打赢，做到守土有责、守土尽责。

打好防范化解重大风险攻坚战。有效防控金融风险，严厉打击非法集资、违规融资担保，完善地方金融监管体制，厘清监管责任，健全风险防范监测预警和早期干预机制，确保不发生系统性、区域性金融风险。有效防控地方债务风险，全面开展调查摸底和风险排查处置，一手抓存量化解，充分利用国家政策窗口期，分类处置存量政府债务；一手抓增量控制，坚决不能再产生新的隐性债务。有效防控养老保险支付风险，开展专项检查，运用信息化和科技手段加强监管，优化支出结构，确保养老金按时足额发放。有效防控安全生产风险，严格落实安全生产责任制，聚焦油气管网、危险化学品、交通运输、人员密集场所等重点行业、领域，全面开展安全生产事故隐患大排查大整治攻坚行动，确保不发生重特大事故，事故起数和死亡人数双下降；大力创建国家食品安全示范城市，强化食品药品全过程监管和食品安全追溯体系建设，食品抽检合格率达到95%以上，保证群众舌尖上的安全。有效防控社会风险，健全重大决策社会稳定风险评估机制，加强信访矛盾排查化解；全力开展扫黑除恶专项斗争，以零容忍态度严打黑恶势力，严惩背后“保护伞”“关系网”；加快构建以“雪亮工程”为重点的社会治安防控体系，推进更高质量平安秦皇岛建设。

打好精准脱贫攻坚战。立足青龙主战场，巩固脱贫成果，确保高质量通过国家验收。落实好扶贫攻坚“六个精准”基本要求，保持组织领导力度不减、帮扶支持力度不减、推进落实力度不减、扶贫政策不变。制定扶贫开发巩固提升三年规划，持续发展扶贫产业，培育壮大龙头企业，强化就业扶持，实现稳定增收。区别致贫原因，落实兜底保障政策，构建“四重医疗保障”体系，持续补齐基础设施、公共服务等突出短板。全面完成易地扶贫搬迁安置任务。继续加大帮扶力度，注重扶贫同扶志、扶智相结合，激发贫困群众稳定脱贫的内生动力。开展扶贫领域腐败和作风问题专项治理，加强扶贫项目资金管理，严肃查处违法违纪行为。

打好污染防治攻坚战。强化污染防治和生态建设薄弱环节，

探索建立主要污染物排放总量控制制度，实施大气、水、土壤污染防治三年作战计划，朝着京津冀城市群生态标兵城市目标持续发力。深化“蓝天行动”，铁腕推进大气污染综合治理，开展工业企业提标改造和深度治理，推动城区内高污染企业退城进郊，彻底整治“散乱污”企业，实施扬尘精细化管控，强化港口、船舶、重载车辆及挥发性有机物污染治理，科学实施错峰生产、错峰运输，淘汰35蒸吨及以下燃煤锅炉，加强劣质散煤管控，全面加强城乡散煤替代，稳步推进冬季清洁取暖工程，坚决打赢“蓝天保卫战”。实施“碧水行动”，巩固水污染防治成果，深入落实河长制，实施一河一策，建设饮用水水源地保护区隔离防护工程，加大坑塘河渠、城市黑臭水体等综合整治，推进城市污水处理设施升级改造，污水处理率达到95%以上，逐步实现出水污染物浓度达到受纳水体环境功能区标准，确保主要河流省考核断面水质优良比例提高10个百分点以上。全面落实湾长制，强化北戴河近岸海域治理，确保海岸沙滩秩序明显好转，海水浴场一类水质比例稳定提高。开展“净土行动”，全面完成农用地土壤污染状况详查，加快推进22个露天矿山整治，逐步实现矿山有序退出，支持领先生物、远丰科技等一批生态修复专业化企业加快发展。加强水资源保护，实施地下水超采综合治理项目。推进国土绿化三年行动计划，实施造林绿化十大工程，完成造林40万亩，建设海滨国家森林公园，森林覆盖率提高3个百分点，确保成功创建国家森林城市。加大对黄金海岸自然保护区遗留问题处置力度。推进柳江国家地质公园申报世界地质公园工作。倾力办好省第二届园博会，将栖云山片区打造成为城市新名片、经济新亮点、休闲新空间。严惩破坏生态环境行为，严守生态保护红线，加强环境执法、监测标准化建设，使监管铁拳攥得更紧、威力更大。

（二）着力构建高质量的现代化产业体系。紧紧扭住经济建设这个中心，把现代化产业体系作为建设现代化经济体系的重要支撑，优化存量资源配置、扩大优质增量供给，确保经济在质的大幅提升中实现量的有效增长。

强化项目建设支撑。坚持以项目扩投资、增后劲、优结构，突出先进制造业、现代服务业、战略性新兴产业等重点领域，突出绿色投资，突出有效投资，促进新旧动能转换，努力抢占现代高效高质产业制高点。完善领导分包和协调推进、考核奖惩等机制，发挥好投资促进和企业服务中心办理组作用，组织好项目集中开工、定期调度考核等活动，盯住盯紧9项省级、100项市级重点项目，确保按计划落地开工建设，尽快投产达效。准确把握国家政策导向和动态形势，抓紧谋划储备一批对经济发展、结构调整具有重大拉动作用的项目，建立梯次跟进的项目储备库，加快形成与建立现代化产业体系相适应的项目投资新格局。正确处理市场主导和政府引导的关系，完善鼓励民间投资的政策措施，激发民间投资活力。全面落实重点企业联系服务机制，扶优做强骨干企业，培育壮大一批中小微企业，年内新增规模以上工业企业40家，新增市场主体2万户，打牢实体经济发展基础。

加快产业转型升级。持续深化供给侧结构性改革，在“破”“立”“降”上下功夫。更加严格执行质量、环保、能耗、安全等法规标准，加大各种违法违规行为治理力度，倒逼钢铁、玻璃、水泥等行业向高端、优质、新兴产业转型发展。支持首秦公司产能退出，实现腾笼换鸟、转型升级。千方百计降成本，开展“降费减负”专项行动，落实“降税减证提标”三项措施。推进制造业强市战略，落实我市“十三五”工业转型发展规划，实施百项重点技改项目计划，下大力推进哈电集团燃气轮机、威卡威新能源汽车等一批重大项目，推动重点行业提质升级。落实省战略性新兴产业发展三年行动计划，以物联网、高端装备制造等领域为主攻方向，实施高技术产业化等工程，加快发展电子信息、新材料、新能源等产业，大力发展数字经济、共享经济、现代供应链等新业态新模式。积极发展“大智移云”产业，推进河北广电网络智慧产业平台项目，建设京津冀健康大数据试验区。深化与中船重工第七一四研究所等机构的战略合作，发展海洋生物、海洋高端装备制造等产业。积极发展现代服务业，推动服务业扩规模提质量。整合提升临港物流园区，推进大宗商品交易中心建设；加快发展大健康、会议会展等产业。努力扩大消费需求，发展数字家庭、智慧养老、远程定制、体验分享等消费新模式；支持电子商务创业园区建设，实施电子商务进社区工程。全力推进军民融合深度发展，年内军民融合企业达到50家。

切实发挥各级开发区平台作用。深化开发区体制机制、投融资和聘任制改革，建立落后淘汰机制，推动开发区提档升级。持续打好开发区发展十大攻坚战，加强承载能力建设，每个开发区都搭建一个投融资平台，建设一批水、电、路、讯、暖等基础设施，储备1000亩以上的建设用地，引进一批重要产业项目。以中信戴卡为龙头，打造全球最大铝制轻量化汽车零配件基地，实现汽车铝制零配件产品全覆盖。推动正大食品产业园加快发展。支持秦皇岛经济技术开发区开展“第三次创业”，加快打造京秦创新合作发展示范区、大数据产业基地。实质性运营开发区集团，引导秦皇岛经济技术开发区与县区开发区（园区）统筹规划建设、要素整合和项目摆放，培育“专精特新”中小企业和产业集群，打造优势互补、互利共赢的发展共同体，带动县域经济进一步增比进位，力争省级以上开发区主营业务收入增长20%。

筑牢全国质量强市示范市品牌。以通过全国质量强市示范市国家验收为契机，持续开展质量提升十个专项行动，着力解决重点行业和领域质量发展中的关键和共性问题，切实增强质量竞争力。推动实施品牌战略，深入实施新产品开发、名牌产品培育工程，开展国家和省级知名品牌创建示范区建设，推进多种形式的品牌发展模式，提高我市品牌的知名度、美誉度和影响力。弘扬工匠精神，开展重点行业国内外标准比对行动，鼓励支持制造企业在产品开发、外观设计、产品包装、市场营销等方面加强创新，打造经济社会全覆盖的新型标准体系。落实质量资助和激励制度，完善质量信用分级分类管理和失信“黑名单”制度，促进标准、质量、品牌、信誉联动提升，努力使“秦皇岛制造”更具品牌价值，“秦皇岛标准”更有市场话语权。

（三）推进一流国际旅游城市建设。把一流国际旅游城市作为建设国际化城市的突破口，统筹规划引领，推动城市发展迈向更高层次。

突出高水平规划引领。立足建设一流国际旅游城市定位，坚持以城定港、港产城融合发展思路，完成新一轮城市总体规划编制、土地利用总体规划和秦皇岛港口总体规划调编，加快实现“多规合一”。高标准制定国际旅游城市总体规划，制定落实建设国际旅游城市的实施意见，加快形成层次完善、结构合理、体现国际一流要求的旅游规划体系。加强滨海核心区规划管理，严格控制建筑体量、色彩及建筑风格，积极塑造国际旅游城市特色。办好首届河北国际城市规划设计大赛，借力提升城市规划水平。

加快旅游业提档升级。巩固第二届省旅发大会成果，不断完善全域旅游产品、旅游服务和营销体系，尽快形成“全市域、全季节、全产业、全方位”的旅游发展格局。办好首届市旅发大会，支持北戴河改造提升低端民宿、保护利用老别墅、加强沙滩管护，在戴河景观带综合开发、旅游文化深度融合等方面示范突破，实施一批四季型、融合性旅游项目，率先建成国家全域旅游示范区。加强旅游项目建设和旅游业态引进，推动中保绿都心乐园、碣石国家公园、板厂峪、祖山等景区“五一”前投入运营，冰塘峪、背牛顶、荣盛康养度假区等景区暑期前呈现新面貌；推动北京佳龙集团文化旅游度假区、法国PuyDuFou主题公园等项目签约落地，以关城文化、滨海休闲旅游品牌的国际化，提升旅游产品的国际品位和影响力。力争旅游总收入增长25%以上。

全面提升旅游服务环境。牢固树立“以游客为中心”的服务理念，重拳整治旅游市场秩序，健全旅游综合执法长效机制，坚决打击漫天要价、欺客宰客行为，杜绝低端恶性竞争。坚持“处处皆景观”“时时有服务”“行行+旅游”，着力提高市民素质和涉旅人员服务意识。实施国际品牌入秦工程，加快茂业中心、万豪酒店等重大商业旅游设施建设，提升整体消费档次和水平。实施传统品牌提升推广工程，开展美食、旅游商品设计大赛，推进特色门店、特色街区建设，每个县区至少建成一条特色餐饮街区。鼓励引导开发体现秦皇岛自然历史文化特色的旅游商品。加速布局“互联网+旅游”，扎实开展好旅游基础设施升级年主题活动，完善交通、餐饮、住宿、厕所等配套设施，在城市标识、标牌和咨询服务中广泛运用国际性语言，全链条提升旅游综合承载服务水平，推动旅游业全方位与国际标准看齐对标。

积极推动国际旅游港建设。加强与河港集团对接合作，邀请国际一流规划团队，高水平开展国际旅游港战略研究，加快制定定位准确、路径清晰、时序合理的路线图和时间表，力争5月底形成初步研究成果。启动西港新城区、港口旅游、邮轮港口的建设工作，争取年内开通渤海湾重要城市海上航线。充分利用好京津冀144小时过境免签政策，设计针对国际游客的个性化旅游线路和服务方案，吸引邮轮将我市作为停靠目的地，让更多国际游客来到秦皇岛、畅游秦皇岛。

着力构建完善的交通体系。优化调整市“十三五”综合交通运输发展规划暨推进京津冀协同发展交通一体化规划，实施京秦高速公路北戴河新区支线、唐秦高速、102国道市区绕城通道等重大工程，新建、改建旅游公路300公里，推进秦皇岛北戴河机场改扩建工程，打造集海陆空港一体的全国综合性交通枢纽，努力为秦皇岛旅游装上“风火轮”，插上“双飞翼”。

（四）坚定不移建设创新型城市。坚持把创新作为第一动力，深入实施创新驱动发展战略，制定实施加快推进科技创新的政策措施和科技创新三年行动计划，加快将我市打造成创新高地。

狠抓创新市场主体发展。实施高新技术企业双倍增计划和科技型中小企业成长计划，引导科技企业孵化器健康发展，培育壮大产业技术创新联盟，支持驻秦院校围绕我市主导产业组织技术攻关，推动产学研用有机结合。充分发挥秦皇岛银行科技支行作用，做大科技型企业应急转贷资金和中小微企业贷款风险补偿金规模，重点向科技型企业倾斜，加快壮大一批率先使用新科技、发明新产品、满足新需求、创造新商业模式的领军企业，年内新增高新技术企业25家以上、科技型中小企业400家以上。推动企业与高校、科研院所成立重点实验室、工程技术研究中心、产业技术研究院等科技创新研发机构，努力在技术创新和关键共性技术研发上有新突破。健全完善财政资金作引导、企业资金为主体、社会资本积极参与的多元创新投资体系，年内研发经费支出占生产总值比重提高到1.5%。

狠抓创新平台培育。完善中小企业技术创新社会化服务体系，支持企业建设企业技术中心、工业设计中心、制造业创新中心等高端创新平台。发挥好驻秦高校、院士工作站等科研机构作用，利用休疗机构改革契机，积极吸引域外大学、研究机构在我市设立研发中心或分支机构。助推秦皇岛高新区扩区发展，推进海港区省级创新型县区试点建设。深化与深圳市工业设计协会合作，打造工业设计“四个一”平台。年内新增市级以上创新平台30家以上。

狠抓创新人才队伍建设。实施顶尖人才、科技领军人才工程和港城英才计划，推进人才安居工程，年内推出600套人才公寓，留住用好本地人才，积极吸引高端人才、创新团队来秦发展。切实发挥好企业家作为财富创造者和创新实践者的作用，完善激励机制、容错机制和帮扶机制，建立企业家与政府微信平台，聘请优秀企业家做经济发展顾问；加大对侵犯企业家合法权益案件的打击力度，激发和保护企业家创新创业精神，培育壮大优秀企业家队伍。认真落实鼓励和支持科技创新的政策措施，打通人才评价、使用、流动、发挥作用的体制机制障碍，营造尊重人才、鼓励创新的浓厚氛围。

（五）毫不动摇深化改革、扩大开放。以改革开放40周年为新起点，实施更高水平的开放、更大力度的改革，用好“两个市场”“两种资源”，加快形成全方位深层次立体式开放新格局。

突出改革重点。深化“放管服”改革，加快市县两级行政审批局规范运行，与投资促进和企业服务中心协同配合，共同服务好企业和群众。做好省政府部门70项行政许可事项取消下放衔接工作，以县区“点菜”方式继续下放一批市级行政权力，全面推广“一章制”审批。深化商事制度改革，推进“多证合一”扩容升级。完善国有资产监管体制，加快完成市直部门所属企业注销改制脱钩，积极发展混合所有制经济。深化投融资体制改革，减少政府审批核准，充分激发社会投资潜力。深化财税金融改革，全面深化绩效预算管理改革，落实好环保税改革。实施上市企业培育计划，新增多层次资本市场上市企业20家，至少1家企业在主板上市。深化科技体制改革以及科技投入机制、成果转化收益等改革，加强知识产权保护。深化以城市管理为重点的综合执法体制改革。完成好全国养老服务业综合改革试点、全省创新城市交通综合治理等国家、省赋予的改革试点任务，发挥好示范带动作用。

提高融入京津冀协同发展的层次。以北戴河生命健康产业创新示范区建设为“牛鼻子”，加快对接京津项目落地生根、开花结果，努力在医疗新技术、新药品、新器械审批准入和健康产业对外开放等方面实现突破，确保生物产业孵化器入园企业达15家左右，新开工项目10个以上。推进国家健康大数据应用中心建设。深化“1+1”对接机制，进一步推进与北京海淀区、西城区等重点区域全面合作，积极吸纳北京、天津医疗卫生、文化教育、工业企业等行业产业疏解外溢；推进与北京体育大学合作，推动足球、篮球、冰球运动学院落户，加快特色“体育小镇”建设；推进国家康复辅助器具产业综合创新试点建设。

推动形成全面开放新格局。制定实施加快形成新时代对外开放新格局的政策措施。成立自由贸易港研究推进机构，编制自由贸易港中长期规划，推动港城深度融合、港口转型发展。强力实施精准招商，健全产业链招商指导目录，分级完善招商规划，利用“5·18”经洽会等重要平台，开展多种形式的招商活动，引进龙头项目和产业链关键项目；将东北地区作为战略重点，制定优惠政策，吸引装备制造等先进产业要素向我市转移；全面实行准入前国民待遇加负面清单管理模式，大幅放宽市场准入。提升对外贸易水平，紧抓“一带一路”、中蒙俄经济走廊建设等机遇，建设国家综合保税区等平台，服务好秦仁航线、达沃航线，争取开通更多国际贸易航线；进一步提升口岸建设及查验设施整体水平，争取更多的货物能在秦皇岛港通关上岸；扎实推进昌黎水产品出口质量安全示范区建设，谋划建设板栗出口产品质量安全示范区，积极发展跨境电子商务；支持企业拓展“一带一路”沿线国家和地区市场，开展工程承包和国际产能合作；鼓励企业并购国外资源、先进技术、研发团队、营销网络，带动我市经济嵌入全球产业链、价值链。

大力优化营商环境。认真落实省《优化营商环境条例》，制定我市政策措施，开展招商引资和服务企业竞赛，营造良好的法治环境、市场环境、社会环境，持续激发市场活力和社会创造力。深化“互联网+政务服务”建设，优化市长热线等便民服务体系。深化“最多跑一次”改革，力争办理事项增加至500项，继续落实好“首问负责制”，让信息多跑路、群众少跑腿。重拳治理乱收费、乱摊派等行为，全面推行“双随机一公开”监管，全面清理“红顶中介”，狠抓一批破坏营商环境的典型，通过优化服务增强企业竞争力。坚持“两个毫不动摇”，下决心破除信贷、人才等方面的歧视性限制和隐性障碍。做好各项优惠政策的宣传落实，切实打通“最后一公里”，让企业第一时间知晓、利用。积极开展“创新创业、服务发展服务民生”活动，瞄准群众和企业需求，提供高质量、高效率、全方位的服务，以权力的“减法”、服务的“加法”，让企业和群众办事更方便、更快捷。

（六）深入实施乡村振兴战略。按照产业兴旺、生态宜居、乡风文明、治理有效、生活富裕的总要求，推进农业农村优先发展，努力让农业成为有奔头的产业，让农民成为有吸引力的职业，让农村成为安居乐业的美丽家园。

深化农业供给侧结构性改革。制定实施农业供给侧结构性改革三年行动计划，实施农产品加工倍增计划，加快农业产业综合体建设，年内每个县都建设一个现代农业园区。推进“互联网+”现代农业发展和农村一二三产业融合发展，积极培育休闲农业、乡村旅游等新业态。坚持质量兴农、绿色兴农，积极推进国家级农产品质量安全市创建，严格禁止、坚决打击使用违禁农兽药不法行为，年内农业标准化率达到55%以上，化肥、农药使用量分别降低10%和5%以上，规模养殖场全部配建粪污处理设施，畜禽养殖废弃物资源化利用率达到75%以上。巩固提升粮食综合生产能力，改善农田灌溉面积14万亩。积极创建特色农业品牌，推动昌黎葡萄、卢龙甘薯、青龙板栗和中药材、抚宁生姜和生猪、山海关大樱桃等特色品牌产生更大经济效益，年内无公害、绿色、有机农产品认证达到300个，打造全域绿色有机农产品生产市。

扎实开展强村富民行动。完善农村承包地“三权分置”制度，深化农村集体产权制度改革，加快农村水价综合改革，完成供销合作社“联合社治理机制创新”全国专项试点工作，让农民获得更多的改革红利。盘活农村各类资产资源，积极发展土地、农宅、资金、资产等多种形式股份合作，持续壮大农村集体经济。积极培育合作社、家庭农场、农业企业、农业产业化联合体等新型农业经营主体，建立完善利益链接机制，促进小农户和现代农业发展有机衔接，实现农民多重增收。加强职业技能培训，切实提高农民致富增收能力，加快农民职业化进程。

大幅改善农村生产生活环境。以建设美丽宜居乡村为导向，以“厕所革命”、垃圾污水治理和村容村貌整治等专项行动为重点，推进农村人居环境整治三年行动，三年全部完成农村厕所改造任务，年内农村改厕20万座以上，农村垃圾处理率达到95%以上。完善农村基础设施，全面完成107座小塘坝防洪隐患治理任务。健全自治、法治、德治相结合的乡村治理体系，大力推进健康乡村、平安乡村建设。加强农村思想道德教育，推动农村移风易俗，培育文明乡风、良好家风、淳朴民风。建立健全实施乡村振兴战略财政投入保障机制，加大金融服务“三农”力度，引导城市资金、技术、人才等现代要素向农村流动，培养一支懂农业、爱农村、爱农民的“三农”工作队伍。

（七）稳步推进新型城镇化建设。坚持以人为本、规划引领、因地制宜，统筹规划建设管理，加快形成城乡区域协调发展新格局。

着力巩固提升文明城市创建成果。把全国文明城市的新高度变成新起点，开展城市面貌综合提升三年攻坚行动，今年重点打造人民广场、迎宾路等重要路段节点景观和绿化亮化改造提升精品。坚持片长制、路长制等有效机制，常态化推进“走遍秦皇岛”“双违”整治、“全民洗城”行动，开展“访千楼万家”等暖心行动，持续加强交通系统治理、背街小巷管理等群众关注问题的整治，开展无障碍设施建设专项行动，建设标准化农贸市场，努力消除马路市场、占道经营等乱象，全面开展“狗患”治理，提高城市管理水平。深化精细化环卫作业，城市垃圾无害化处理率达到97%以上。开展“厕所革命”，新增城区标准公共厕所160座、改造提升133座，建立完善公厕建设管理体制机制。扎实精致做好创建国家卫生城市各项工作。深入实施“城市双修”工程，不断提升城市风貌形象，加快恢复城市生态系统调节功能。

着力提升城市承载功能。坚持适度超前、循序渐进，推进海绵城市、地下管廊建设和老旧管网改造。完善北戴河西部水厂后续工程，建设水质检测中心，协调推进中俄东线输气管线配套工程，谋划新能源热源厂建设，推进光热、光伏、风电等供暖试点。实施火车站北站房、北广场及周边路网建设，改造拓宽河北大街、和平大街中段，实施海滨路东西延伸工程，让道南片区道路出行不再“难”；实施峨眉山路北延伸等工程，逐步打通城市“断头路”，使城区交通环境有新的跃升。

着力提升城镇发展水平。发挥县城节点作用，引导生产要素、优势资源向县城集中，促进农业转移人口市民化和产城教融合发展。积极稳妥地推进行政区划局部调整，鼓励开展乡改镇、镇改办事处、村改居工作，确保常住人口城镇化率突破60%。积极推动海港区申报省级新型城镇化综合试点，打造一批产业品牌化、园镇一体化的特色小镇。

（八）更好满足人民群众美好生活需要。坚持将人民对美好生活的向往作为我们的奋斗目标，尽力而为、量力而行，以为民实事十大工程为抓手，多谋民生之利，多解民生之忧，让全市人民获得感更充实、幸福感更真实、安全感更踏实。

提高社保就业质量。深入推进全民参保计划，全面落实企业养老保险省级统筹和机关事业单位养老保险制度改革措施，健全失业保险金标准调整机制。深化医保支付方式改革，全面推行以按病种付费为主的多元复合付费方式。城镇、农村低保标准分别提高到每人每年7200元和4000元。深入落实高校毕业生就业促进计划、创业引领计划，扎实做好产业分流职工、就业困难人员和退役军人等重点群体安置工作。坚持创业引领就业，加快建设全省最高水平的公共就业实训基地，完善创业担保贷款政策，支持农民工等人员返乡创业，实施农民工技能培训1万人，新增城镇就业5万人。

满足群众住房需求。保持房地产市场调控政策连续性和稳定性，支持专业化、机构化住房租赁企业发展，建立多主体供应、多渠道保障、租购并举的住房制度。实施新的三年棚户区改造攻坚计划，年内新开工8000套。以被国家住建部列为全国老旧小区改造试点为契机，探索建立老旧小区改造长效机制，提升物业管理服务水平。实施“征拆难”“入住难”攻坚行动，更高水平地解决好房地产开发领域遗留问题。对不良建筑、房地产企业实施惩戒，建立淘汰曝光机制，促进建筑、房地产领域健康发展。

提升社会事业水平。优先发展教育事业，努力破解“大班额”“学区热”和“入托贵”等突出问题，每年安排专项资金1亿元，实施教育资源优化提升三年行动计划，年内新改扩建公办幼儿园13所、中小学15所、高中学校2所，撤并教学点80个；积极组建教育集团；统筹推进高中教育普及攻坚和高等职业教育创新发展；支持燕山大学、东北大学秦皇岛分校“双一流”建设，河北科技师范学院筹建河北海洋大学。深化医药卫生体制改革，9个医联体加快规范运营步伐，26家公立医院全部建立现代医院管理制度，逐步建立分级诊疗体系；每年安排1亿元专项资金，实施基层医疗机构达标三年计划，年内新建、改造乡镇卫生院和社区卫生服务中心25所；启动市工人医院、市第三医院改造工程；推动中医药事业发展。积极稳妥有序实施全面两孩政策。全面构建现代公共文化服务体系，推进市博物馆建设；建设城市书房、“城市家具”、沙滩书吧，丰富群众文化生活；落实全民健身计划，为200个行政村（社区）安装健身路径；发展冰雪、帆船（帆板）等户外运动，组织不少于2万名中小学生免费参加冰雪、帆船（帆板）运动体验和培训。办好国际马拉松等重要赛事，支持中式八球运动推广，做大做强体育产业，在全省率先建设文化体育强市。

加强社会治理创新。创新社会治理机制，积极探索共建共治共享的小区管理新机制、新模式。建立健全镇街、村居的权力责任清单，培育和发挥社会组织的专业化参与作用，加强社会诚信体系建设，加快构建以社区为平台、社会组织为依托、社会专业人才为支撑的“三社联动”机制。推进应急保障体系、市级防灾减灾救灾物资储备库和突发事件预警信息发布体系建设，提高公共突发事件应急反应和保障能力。

深入实施“两河战略”，高标准做好旅游旺季服务保障工作，自觉当好首都政治“护城河”。加强国防教育、国防动员和后备力量建设，支持部队推进新时代强军事业，深入开展双拥共建活动，巩固军政军民团结的好局面。支持工会、共青团、妇联、科协等人民团体发挥桥梁纽带作用，做好新闻出版、广播电视、老龄、残疾人、妇女儿童、气象、防震减灾、民族宗教、外事侨务、人民防空、邮政通信、审计、统计、地方志、档案和援疆等工作，推动各项事业取得新进步。

各位代表，征程万里风正劲，重任千钧再扬鞭。只要我们咬定青山不放松，撸起袖子加油干，就一定能够推动党的十九大精神和习近平新时代中国特色社会主义思想在我市落地生根、开花结果，谱写好伟大中国梦的秦皇岛篇章！

四、全面加强政府自身建设

打铁必须自身硬。我们将牢记职责使命，加强政府改革创新，以更高标准、更严要求、更实作风不断提升履职能力和水平。

（一）坚定正确政治方向。深入学习贯彻党的十九大精神，着力在学懂、弄通、做实上下功夫。牢固树立“四个意识”，把维护以习近平同志为核心的党中央权威和集中统一领导作为最高政治原则和根本政治规矩，始终在政治立场、政治方向、政治原则、政治道路上同党中央保持高度一致，做到党中央提倡的坚决响应、党中央决定的坚决执行、党中央禁止的坚决不做。推进“两学一做”学习教育常态化制度化，扎实开展“不忘初心、牢记使命”主题教育，坚持以习近平新时代中国特色社会主义思想武装头脑、指导实践、推动工作，全面落实中央、省委省政府和市委部署要求，以坚定的思想自觉、政治自觉、行动自觉努力开创新时代政府工作新局面。

（二）加快法治政府建设。认真落实法治政府建设实施纲要，把法治精神、法治思维和法治方式贯穿政府工作全过程，严格执行专家论证、风险评估、合法性审查等重大行政决策法定程序，推进依法、科学、民主决策。加强重点领域立法，深化行政执法三项制度改革，严格规范公正文明执法。支持检察机关公益诉讼，深化行政执法与刑事司法衔接机制建设。自觉接受人大法律监督、工作监督和政协民主监督，广泛听取民主党派、工商联、无党派人士、人民团体意见。推进政务决策、执行、管理、服务和结果全过程公开，让权力在阳光下运行。

（三）驰而不息转变作风。严格落实中央八项规定精神，持续开展纠正“四风”和作风纪律专项整治。深入推行工作日志、“五到四从四多”“两式两化”“四位一体”联动督查等制度，大力弘扬“站在排头不让，扛着红旗不放”的精神，引导各级党员干部既要有功成不必在我的胸怀境界，更要有功成必定有我的使命担当。下大力狠刹不作为、乱作为、“吃拿卡要”隐形变种等不正之风，真正做到一抓到底，见人见事见结果。大兴调查研究之风，深入基层、深入一线，找准短板和症结，精准破解发展中的难点。

（四）持之以恒正风肃纪。认真落实全面从严治党主体责任和“一岗双责”，坚持把纪律和规矩挺在前面，切实把党风廉政建设和反腐败工作落到实处。坚持源头防范，加大行政审批、资源开发、土地出让、工程招投标及公共财政支出等领域的监管力度，铲除滋生腐败土壤。下决心狠治“小范围、小活动、小表示、小变通”等微腐败行为。严查重点领域和关键岗位腐败，严惩群众身边腐败问题，做到无禁区、全覆盖、零容忍。坚持严管和厚爱、激励和约束并重，认真落实奖优罚劣和容错免责机制，旗帜鲜明为那些敢于担当、踏实做事、不谋私利的干部撑腰鼓劲，让敢担当者挺直腰杆，让无为者无处立足。

各位代表，使命呼唤担当，实干赢得未来。秦皇岛发展已站在新的起点上，让我们紧密团结在以习近平同志为核心的党中央周围，在省委省政府和市委的坚强领导下，不忘初心、牢记使命，勇于担当、砥砺前行，为决胜全面建成高质量小康社会，加快建设新时代沿海强市、美丽港城和国际化城市而努力奋斗!
